# The Effect of Hip Arthroplasty on Gait Function: Comparison of Ceramic‐On‐Ceramic Hip Resurfacing, Metal‐On‐Metal Hip Resurfacing, and Total Hip Arthroplasty

**DOI:** 10.1002/jor.70112

**Published:** 2026-01-07

**Authors:** Dylan Leon, Amy Maslivec, Brogan Guest, Natasha Allott, Justin Cobb

**Affiliations:** ^1^ Imperial College London Sir Michael Uren Hub London UK; ^2^ Embody Orthopaedic Limited, Sir Michael Uren Hub London UK

**Keywords:** gait, ground reaction force, hip resurfacing arthroplasty, MET score, total hip arthroplasty

## Abstract

Ceramic‐on‐ceramic hip resurfacing arthroplasty (CoC‐HRA) has been developed to eliminate metal ion concerns which have been associated with metal‐on‐metal hip resurfacing arthroplasty (MoM‐HRA) while maintaining similar functionality. The aim of the study was to examine gait function pre‐ and postoperatively between CoC‐HRA, MoM‐HRA, and THA using subjective and objective measures with comparison to a healthy control group. Nineteen unilateral CoC‐HRA, 19 unilateral MoM‐HRA, and 18 unilateral THA gender, age, and BMI matched participants completed patient‐reported outcome measures (PROMs) (Oxford hip score [OHS] and metabolic equivalence of task score [MET]) and underwent gait analysis on an instrumented treadmill, preoperatively (2–8 weeks) and then postoperatively (40–52 weeks). Spatiotemporal measures and vertical ground reaction forces (GRF) were recorded. Statistical parametric mapping was used to detect differences in GRF between affected and nonaffected leg and to healthy controls. Preoperatively, there were no differences between groups in PROMs or objective measures. All groups showed an improved OHS postoperatively with only CoC‐HRA and MoM‐HRA demonstrating significant increase in MET. Postoperatively, TWS in both HRA groups improved with no difference to CON while THA was unable to demonstrate improvements. Postoperatively, at 6.5 km/h, THA demonstrated an asymmetric GRF profile, whereas CoC‐HRA and MoM‐HRA showed no differences between legs. In comparison of the affected leg GRF, THA demonstrated a weaker push off when compared to both resurfacing groups and CON. CoC‐HRA and MoM‐HRA showed no significant differences to CON. CoC‐HRA emerges as a potential alternative to MoM‐HRA, effectively addressing metal ion release concerns while retaining similar functional benefits.

## 1 Introduction

End‐stage hip osteoarthritis (OA) negatively impacts physical function and quality of life. Due to an aging population, the prevalence of hip OA is rising [1], with hip arthroplasty being an inevitable option. Total hip arthroplasty (THA) accounts for over 98% of all primary hip arthroplasties in the United Kingdom [2] and is considered an effective procedure that offers long‐term survivorship, amelioration of joint pain, and improvement of function [2, 3, 4]. However, patient expectations have evolved beyond pain relief and basic functional recovery to include the desire for active lifestyles and the ability to engage in high‐intensity activities. Despite improvements in function from preoperative levels, THA has not been shown to restore gait function to that of healthy controls [5] nor reach the same physical activity levels [5, 6].

Metal‐on‐metal hip resurfacing arthroplasty (MoM‐HRA) is a bone‐conserving alternative to THA developed to better restore hip mechanics by maintaining the shape and structure of the proximal femur, and in turn improved function [7]. Younger patients with OA or those with developmental hip pathology may benefit from MoM‐HRA since its bone‐conserving approach makes inevitable future revision surgery more straightforward. Three randomized controlled studies (RCTs) using gait analysis failed to detect differences between MoM‐HRA and THA patients [8, 9, 10]. However, this was restricted to slow walking speeds. When assessed at faster walking speeds, MoM‐HRA patients demonstrated a more symmetrical gait profile and better hip range of motion compared to THA [8]. MoM‐HRA has been shown to restore gait function to a level similar to healthy controls [8, 9, 10, 11].

Despite better function in MoM‐HRA compared to THA, there are concerns surrounding MoM‐HRA such as excessive wear, increased Chromium and Cobalt ion levels, early implant failure, and related postoperative pain [12]. However, these complications were commonly associated with poorly placed prosthetic components and smaller bone sizes. Therefore, the clinical use of MoM‐HRA has since been restricted to younger men with larger femoral heads [13, 14], resulting in less than 1% of all primary hip arthroplasty being HRA [2].

By changing the metal bearing to ceramic (BIOLOX *delta)*, ceramic‐on‐ceramic hip resurfacing arthroplasty (CoC‐HRA) aims to maintain the positive clinical and functional performance of MoM‐HRA over THA, while avoiding the metal‐on‐metal complications. To date, there is only one study comparing CoC‐HRA to THA, involving exclusively female patients [15], which demonstrated weaker push‐off force and increased gait asymmetry of THA when compared to CoC‐HRA. There is no published data comparing functional outcomes between patients undergoing CoC‐HRA, MoM‐HRA, and THA.

The aim of the study was to examine gait function pre‐ and postoperatively between three hip arthroplasty procedures (CoC‐HRA, MoM‐HRA, and THA) using subjective and objective measures with comparison to a healthy control group. The primary null hypothesis was that there would be no difference in subjective nor objective measures between patient groups. The second null hypothesis was that patient groups would have gait function that was indistinguishable from healthy controls.

## 2 Methods

### 2.1 Patients

In this prospective observational cohort study, 56 male unilateral hip osteoarthritis patients underwent unilateral hip arthroplasty. Nineteen unilateral CoC‐HRA (Embody Orthopaedic Limited, London, UK), 19 unilateral MoM‐HRA (Smith and Nephew orthopaedics, USA), and 18 unilateral THR using a short‐stemmed implant (Furlong Evolution System, JRI Orthopaedics Ltd, Sheffield, UK). Exclusion criteria included body mass index (BMI)  > 40, previous lower limb surgery, recent injury that could affect gait, and any existing neuromuscular conditions. Ethical approval for the study was obtained (ethics reference number 7/EE/0330, 14/NS/1045), and signed consent was obtained from all patients. All procedures were carried out using a posterior approach. There were 17 healthy, asymptomatic, age‐, sex‐, and BMI‐matched participants recruited as controls.

### 2.2 Subjective Outcome Measures

Patient reported outcome measures (Oxford hip score [OHS] and metabolic equivalent of task score [MET]) were completed pre‐ and postoperatively. MET is a measure of physical activity levels and was calculated using a previous method [15].

### 2.3 Gait Protocol

All patients underwent treadmill gait analysis preoperatively (2–8 weeks) and postoperatively (40–53 weeks). Patient gait data were collected using an instrumented treadmill (HP/COSMOS, Hab International, Munich, Germany). Patients began a familiarization period where they were able to walk unassisted on the treadmill at 3 km/h for 5 min. The starting speed was set to 4 km/h, then further increased by increments of 0.5 km/h every 60 s until patients reached their self‐determined top walking speed (TWS). Data were collected during a 20 s period at each walking speed, and the average of 10 steps for each limb was used for further analysis. The short trials at each speed meant that patient fatigue was negligible. Patients were secured into a safety harness, which didn't restrict mobility. Patients were able to stop at any point with stop buttons on the treadmill within reach.

### 2.4 Gait Variables

#### 2.4.1 Spatiotemporal Variables

TWS was determined as the fastest possible walking speed patients were able to achieve before breaking into a run or being limited by discomfort. TWS was normalized to leg length [16]. Spatiotemporal variables: step length of the operated limb, cadence, contact time, and step width were recorded. To correct for leg length discrepancies, step length was normalized postcollection [16].

#### 2.4.2 Ground Reaction Force (GRF) Profile

Vertical GRF was collected with three variables for analysis: maximum weight acceptance, midstance support, and maximum push‐off. GRFs were normalized to body weight and were time‐normalized to 100% of the stance phase of the gait cycle. GRF of affected‐ and nonaffected limb of patients' groups were analyzed to assess for asymmetry, and GRF of patients affected side postoperatively and control group were analyzed to determine differences between groups. GRF profiles are reported at 5 km/h preoperatively and at speeds of 5 and 6.5 km/h postoperatively, as this was the highest common walking speed achieved by patients.

### 2.5 Statistical Analysis

All data were checked for normality. For OHS, Mann–Whitney *U*‐test was used with Bonferroni correction where differences were detected, and independent samples *t*‐tests were used with Bonferroni correction for MET score. Normality of data was confirmed for TWS, GRF, and spatiotemporal gait variables. Therefore, for comparison of the discrete values between the CoC‐HRA, MoM‐HRA, and THA groups pre‐ and postoperatively, a series of paired sample *t*‐tests was used. To compare the differences between all four groups (CoC‐HRA, MoM‐HRA, THA, and CON), a one‐way analysis of variance (ANOVA) followed by post hoc comparisons using Bonferroni corrections (*p* < 0.05) was carried out. Independent samples *t*‐test (between CoC‐HRA, MoM‐HRA, THA, and CON) for statistical parametric mapping (SPM) were performed to compare the GRF in the time‐normalized (0%–100%) stance phase of the gait cycle. The SPM was calculated at each point of the waveform; if it exceeded the critical threshold of < 0.05, it was considered significant in that part of the waveform. An a priori power calculation was performed based on clinically relevant differences in the primary outcome measures: GRF, a between‐group difference of 10% body weight in push‐off force (SD = 7%), for TWS a difference of 1.36 km/h (SD = 0.89), and for MET a difference of 5.4 units (SD = 3.1), with an alpha of 0.05% and 80% power. The achieved sample sizes (CoC‐HRA: *n* = 19, MoM‐HRA: *n* = 19, THA: *n* = 18) exceeded this threshold, confirming that the study was adequately powered to detect statistically and clinically significant differences in both subjective and objective functional outcomes. The study was built as a superiority trial rather than a noninferiority trial due to the inclusion of three comparison groups and the aims of detecting functional advantages of both resurfacing groups over THA while also demonstrating CoC‐HRA is noninferior to MoM‐HRA (Table 1).

**Table 1:** Characteristics of patients and controls (mean [SD]).

	CoC‐HRA	MoM‐HRA	THA	CON	*p*‐value
Age (Years)	61.2 ± 4.4	60.5 ± 8.2	65.3 ± 7.9	63.1 ± 9.5	0.232
Gender M:F	1:0	1:0	1:0	1:0	n/a
BMI	27.7 ± 3.8	27.2 ± 2.8	26.9 ± 3.0	26.5 ± 3.5	0.755
Pre‐op time (weeks)	1.8 ± 1.9	7.7 ± 11.1	6.0 ± 7.2	n/a	0.06
Post‐op time (weeks)	52.7 ± 3.4	39.5 ± 11.1	44.7 ± 35.6	n/a	0.163

## 3 Results

### 3.1 Subjective Outcome Measures

Preoperatively there were no differences in OHS or MET index between groups (CoC‐HRA: 26, MoM‐HRA: 28, THA: 27, H (2) = 1.467, *p* = 0.480) (Table 2). All groups significantly improved OHS postoperatively above the acceptable significance improvement of 14 (CoC‐HRA: 21 [*p* = 0.00], MoM‐HRA:20 [*p* = 0.00], THA:20 [*p* = 0.00]). CoC‐HRA and MoM‐HRA significantly improved MET score pre‐ and postoperatively (CoC‐HRA: 5.3–10.8 [*p* = 0.00], MoM‐HRA: 5.2–9.9 [*p* = 0.001]) whereas THA did not (4.6–5.4 *p* = 0.826). Postoperatively, CoC‐HRA and MoM‐HRA had a significantly higher MET score compared to THA (CoC‐HRA: 10.8 vs. 5.4 [*p* = 0.00], MoM‐HRA: 9.9 vs. 5.4 [*p* = 0.00] but no difference between CoC‐HRA and MoM‐HRA [*p* = 0.330]).

**Table 2:** Patient reported outcome measures for MoM‐HRA, CoC‐HRA, and THA pre‐ and postoperatively.

	OHS		MET	
	Pre‐op	Post‐op	Pre‐op	Post‐op
CoC‐HRA	26 (7–35)^a^	47 (41–48)	5.3 (0–13.2)^a^	10.8 (6.5–17.2)
MoM‐HRA	28 (17–36)^a^	48 (45–48)	5.2 (0–10.4)^a^	9.9 (5.7–17.1)
THA	27 (17–33)^a^	47 (43–48)	4.6 (0–9.6)	5.4 (0–10.2)^b^

*Note:* Data presented is median (range). Significance level was set to *p* < 0.05.

Abbreviations: MET score, metabolic equivalent of task; OHS, Oxford Hip Score.

^a^ Significantly different from pre to post‐op.

^b^ Significantly different when compared to each of the other patient groups.

### 3.2 Spatiotemporal Variables

Preoperatively, there was no difference in TWS score between groups (CoC‐HRA: 5.89, MoM‐HRA: 6.32, THA: 5.94, H[2] = 2.163, *p* = 0.339) (Table 3). CoC‐HRA and MoM‐HRA significantly improved TWS score pre‐ and postoperatively (CoC‐HRA: 5.89–7.12 km/h [*p* = 0.00], MoM‐HRA: 6.32–7.53 km/h [*p* = 0.000]) whereas THA did not (5.94–6.17 km/h *p* = 0.094). Postoperatively, THA had a significantly lower TWS than CoC‐HRA (*p* = 0.004), MoM‐HRA (*p* = 0.00), and CON (*p* = 0.00). There was no difference in TWS postoperatively between CoC‐HRA (7.12 km/h), MoM‐HRA (7.53 km/h), and CON (7.26 km/h) H(2) = 4.183, *p* = 0.123.

**Table 3:** TWS and Spatiotemporal gait variables for CoC‐HRA, MoM‐HRA, and THA at pre‐ and postoperative time points, and healthy controls.

	TWS (km/h)	Step length	Cadence	Contact time	Step width
Coc‐HRA					
Pre‐op	5.89 ± 1.20^a^	0.68 ± 0.05^a^	51.28 ± 3.85^a^	1.54 ± 0.11	0.09 ± 0.02
Post‐op	7.12 ± 0.88	0.73 ± 0.06	48.92 ± 3.16	1.63 ± 0.10	0.08 ± 0.02
MoM‐HRA					
Pre‐op	6.32 ± 0.99^a^	0.68 ± 0.06^a^	50.53 ± 3.88^a^	1.55 ± 0.14	0.08 ± 0.02
Post‐op	7.53 ± 0.89	0.73 ± 0.06	49.09 ± 2.9	1.61 ± 0.08	0.08 ± 0.02
THA					
Pre‐op	5.94 ± 0.94	0.66 ± 0.04^a^	53.05 ± 3.63^a^	1.48 ± 0.09	0.09 ± 0.02
Post‐op	6.17 ± 0.89^b^	0.68 ± 0.04^b^	51.85 ± 3.39^b^	1.53 ± 0.10^b^	0.09 ± 0.02
Control	7.26 ± 0.50	0.73 ± 0.04	49.26 ± 2.67	1.61 ± 0.1	0.08 ± 0.02

*Note:* All spatiotemporal variables were recorded at 5 km/h pre‐ and postoperatively. Significance level was set to *p* < 0.05.

^a^ Significantly different from pre to post‐op.

^b^ Significantly different when compared to each of the other patient groups.

Preoperatively, there was no difference in step length between patient groups *F*(2, 53) = 1.401, *p* = 0.255) (Table 3). THA improved step length pre‐post (*p* = 0.038), MoM‐HRA improved step length pre‐post (*p* = 0.002), and CoC‐HRA improved step length pre‐post (*p* = 0.025). Postoperatively, THA had a shorter step length compared to MoM‐HRA (*p* = 0.023), CoC‐HRA (*p* = 0.001), and CON (*p* = 0.05). There was no difference postoperatively between CoC‐HRA, MoM‐HRA, and CON.

Preoperatively, there were no difference in cadence between patient groups *F*(2, 53) = 2.144, *p* = 0.127) (Table 3). THA did not change cadence from pre‐post (*p* = 0.19), CoC‐HRA reduced cadence from pre‐post (*p* = 0.002), MoM‐HRA reduced cadence from pre‐post (*p* = 0.025). Postoperatively, THA had a higher cadence compared to CoC‐HRA (*p* = 0.029), MoM‐HRA (*p* = 0.046) and CON (*p* = 0.047). There was no difference between CoC‐HRA, MoM‐HRA, and CON.

Preoperatively, there were no difference in contact time between patient groups *F*(2, 53) = 1.1789, *p* = 0.274) (Table 3). THA did not change contact time from pre‐post (*p* = 0.084). CoC‐HRA increased contact time from pre‐post (0.00), MoM‐HRA did not change contact time from pre‐post (*p* = 0.025). Postoperatively, THA had a lower contact time compared to CoC‐HRA (*p* = 0.015), MoM‐HRA (*p* = 0.042), and CON (*p* = 0.004). There was no difference between CoC‐HRA, MoM‐HRA, and CON.

Preoperatively, there was no difference in step width between patient groups *F*(2, 53) = 8.66, *p* = 0.426) (Table 3). THA did not change step width from pre‐post (*p* = 0.401), CoC‐HRA did not change step width from pre‐post (*p* = 0.057), MoM‐HRA did not change step width from pre‐post (*p* = 0.46). There was no difference in step width postoperatively between all groups *F*(3, 69) = 1.241, *p* = 0.301.

### 3.3 GRF Profile

**Figure 1:**
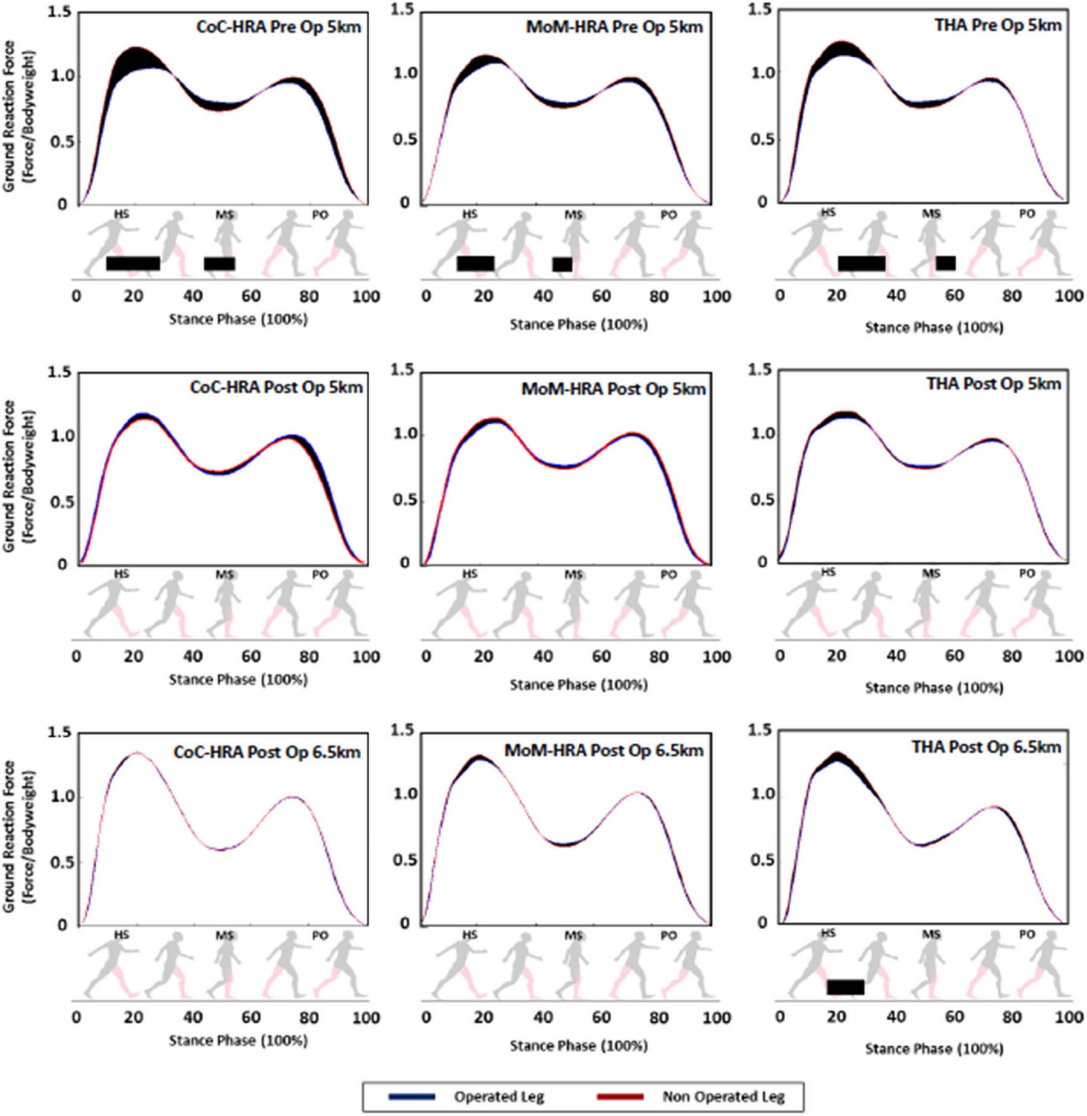
Normalized to body weight: Ground reaction force profile of affected and nonaffected leg at preoperatively 5 kmph, postoperatively 5 kmph, and postoperatively 6.5 kmph for CoC‐HRA, MoM‐HRA, and THA at 5 kmph. SPM results are displayed below the figures and black bars indicate significant difference.

Preoperatively, all three patients groups displayed an asymmetric GRF profile, producing significantly higher GRF of the nonaffected limb compared to the affected limb at heel strike (CoC‐HRA: 14% and 26% [*p* = 0.02], MoM‐HRA: 13% and 22% [*p* = 0.02], THA: 15% and 28% [*p* = 0.01]) and higher force of the affected limb at mid stance (CoC‐HRA: 43% and 58% [*p* = 0.03], MoM‐HRA: 43 and 50 [*p* = 0.03], THA: 56% and 60% [*p* = 0.02]) of the stance phase (Figure 1). Postoperatively, CoC‐HRA and MoM‐HRA displayed a symmetric GRF profile with SPM not detecting any significant differences in GRF between nonaffected and affected limb. THA displayed an asymmetric GRF profile, producing significantly higher GRF of the nonaffected limb compared to the affected limb at heel strike (18% and 28%, [*p* = 0.01]).

**Figure 2:**
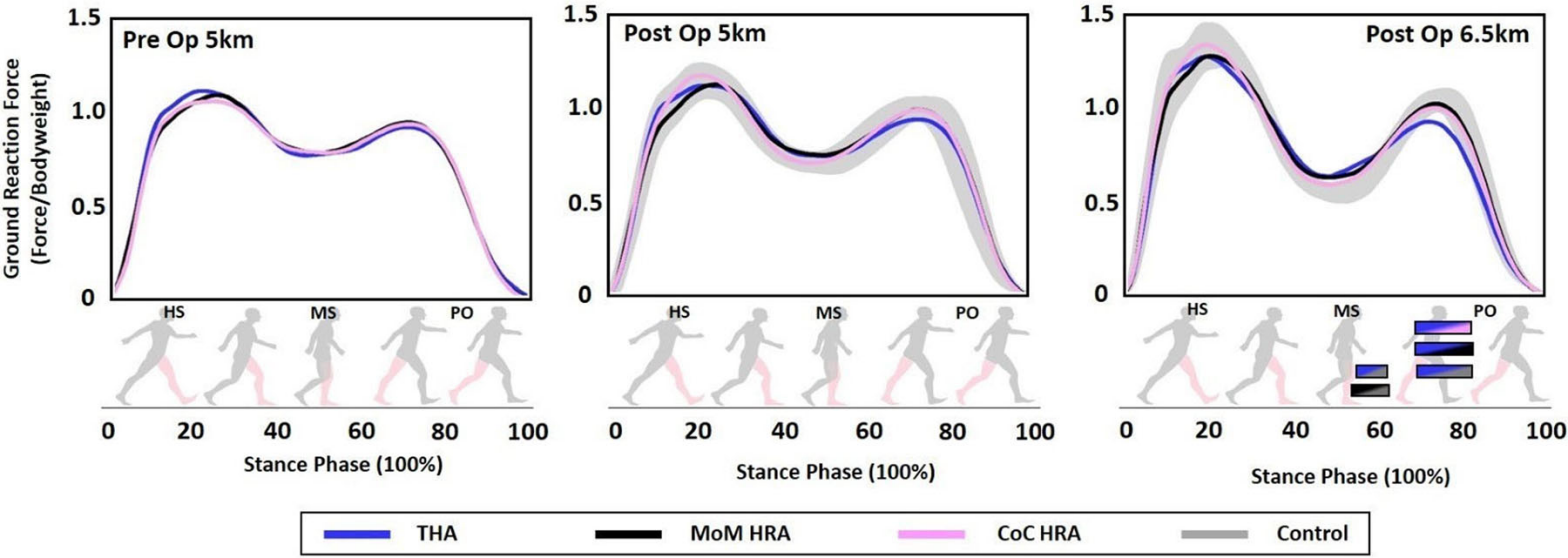
Normalized to body weight: Ground reaction force profiles for CoC‐HRA and MoM‐HRA postoperatively, as well as the normative range (mean ± 1 standard deviation) derived from the control group data.

Comparing the affected leg between groups, preoperatively, no differences were found in the GRF profile between groups (Figure 2). Postoperatively, at walking speeds of 5 km/h, no group differences were found in the GRF profile. At walking speeds of 6.5 km/h, THA showed a significant weaker push of between 70% and 83% (*p* = 0.02) of the stance phase compared to CoC‐HRA, MoM‐HRA, and controls.

## 4 Discussion

This study sought to examine functional outcomes of patients who received either CoC‐HRA, MoM‐HRA, or THA. There was no significant difference across all groups preoperatively in PROMs, TWS, gait asymmetry, or spatiotemporal measures. Both CoC‐HRA and MoM‐HRA significantly improved PROMs, TWS, and restored a symmetrical gait pattern, which were indistinguishable from each other and healthy controls; therefore, both null hypotheses were rejected. In contrast, THA did not improve TWS, maintained deficits in spatiotemporal measures and gait symmetry with reduced push off at faster speeds.

This study utilized subjective measures of function, OHS, and MET scores pre‐ and postoperatively. As expected, all groups improved OHS to maximum scores, which reinforces previously reported ceiling effects of this metric [17]. The MET score was introduced as a self‐reported method of physical activity with normal distribution, recording levels of activity that the OHS cannot detect. A MET score between 5 and 7 indicates moderate intensity activity, while a score greater than 7 suggests high intensity [18]. CoC‐HRA and MoM‐HRA increased activity levels to over 7 METs (CoC‐HRA: 5.3–10.8 METs, MoM‐HRA: 5.2–9.9 METs) whereas THA were unable to (4.6–5.4 METs). Physical activity is an important aspect of health and well‐being with high‐intensity activity associated with long‐term health gains and a reduced risk of morbidity and early mortality [19]. The findings within this study align with previous research, which highlighted the ability of MoM‐HRA to reach high levels of self‐reported activity and return to sports in men [8, 20].

TWS is a validated predictor of functional recovery and ability following hip arthroplasty [21]. In this study, both CoC‐HRA and MoM‐HRA groups demonstrated improvements in TWS from similar preoperative levels (MoM‐HRA: 6.4 vs. 7.4 km/h, CoC‐HRA: 6.3 vs. 7.4 km/h), exceeding the minimal clinically important difference (MCID) of 0.36 km/h [22]. Both HRA groups achieved TWS comparable to healthy controls (7.3 km/h), consistent with previous MoM‐HRA findings of an RCT [8] (7.3 km/h) [11] and (7.5 km/h). However, THA was not able to achieve significant improvements in TWS (5.9–6.2 km/h).

Before surgery, all three groups exhibited antalgic gait patterns characterized by excessive loading due to compensation of the healthy contralateral limb at weight acceptance, which is a common feature of OA gait [23]. In SPM analysis, CoC‐HRA and MoM‐HRA demonstrated improvements in the asymmetrical loading patterns from pre‐ to postoperative stages, with no difference in postoperative GRF loading patterns between affected and nonaffected leg, even at faster speeds. The GRF pattern of these groups was additionally indistinguishable from healthy controls. However, in THA despite restoration of symmetry at slower speeds, faster speeds revealed a reduced push‐off force of the affected leg compared to both HRA groups, reflecting previous studies [8] and [24], at faster speeds. At faster walking speeds, joint stress and movement demands rise, making underlying deficits more apparent as compensatory strategies become less effective. Despite the lack of randomization in the current study, the outcomes mirrored results from gold standard RCTs [8, 9, 10], thereby lending validation to the findings. To date, there is only one published study comparing CoC‐HRA to THA, involving exclusively female patients, which similarly demonstrated reduced push‐off and greater limb asymmetry of THA at higher speeds when compared to CoC‐HRA [15].

In SPM analysis of the affected leg GRF between groups, THA had a weaker push off at higher speeds when compared to CoC‐HRA, MoM‐HRA, and CON. The difference in push‐off may be attributed to the presence of an intramedullary stem stiffening the femur, decreasing weight tolerance, resulting in reduced power generation by the affected leg. In addition, Gerhart et al. [8] further demonstrated reduced postoperative hip range of motion in THA compared to MoM‐HRA which may be due to the smaller femoral head component diameter of THA compared to MoM‐HRA. The difference in TWS and MET index between groups may also be explained by these combined factors.

Postoperatively, THA was unable to demonstrate significant improvements in step length or significant decreases in step cadence. CoC‐HRA and MoM‐HRA were able to improve all spatiotemporal measures (step length, cadence, contact time, and step width) to levels indistinguishable from healthy controls. The differences can be attributed to the persistent abnormal hip loading in postoperative THA patients. HRA patients restore gait function to physiological levels, however, despite improvements in asymmetry for THA patients, abnormal hip loading persists [25]. Increases in step cadence and reduction in step length are compensatory measures adopted by patients with asymmetric gait profiles. The consequence of these changes is a reduced gait speed, hence, providing the biomechanical basis for the reduced TWS in THA patients.

This small study has notable strengths, being the first to investigate functional outcomes of patients undergoing CoC‐HRA compared to MoM‐HRA. The use of objective gait measurements through incremental walking speeds has enabled the detection of gait differences that went unnoticed at lower speeds. All surgeries were performed by the same surgeon using a posterior approach, minimizing operative variation between groups. The inclusion of preoperative data allowed for a direct comparison of improvement between groups, revealing no baseline differences in subjective nor objective measures. All THAs in this cohort were short‐stem implants, which preserve more native bone and previous studies have shown improved gait outcomes compared to long‐stem designs [26]. Using this more favorable implant type as a comparator further reinforces the significance of our findings.

This study has some limitations to consider. First, since this is a prospective observational cohort analysis and not a randomized control trial, there is an inherent selection bias. The resurfacing patient demographic may have higher baseline motivation, lifestyle expectations, and overall health literacy influencing them to opt for the nonconventional resurfacing rather than the THA. This may be a confounding factor contributing to the difference in postoperative functional outcome between these groups, therefore, further studies exploring long‐term postoperative time frames need to be carried out. Second, there is an exclusive focus on male patients due to the contraindication of MoM‐HRA in females. This study utilized the contralateral hip as a reference for symmetry assessment which meant excluding those with any contralateral pathology, limiting the study to participants with monoarticular disease. Individuals with hip OA commonly exhibit symptoms in multiple joints, therefore, this study may not be fully representative of the broader hip OA population. Another limitation was the variation in the timing of preoperative gait analysis and PROMs data collection between groups. However, given the gradual progression of OA, the difference in weeks is unlikely to significantly affect preoperative function. Lastly, it is acknowledged that CoC‐HRA is a relatively novel implant, with the first patient treated in 2017. Although early outcomes have been encouraging, longer‐term data are essential to confirm the durability and clinical performance of this device, which is intended to serve patients for many years. A 5‐year follow‐up report is currently in preparation; however, extended observation periods will be necessary before definitive conclusions can be drawn. In contrast, primary THA is well established, with a reported 25‐year survival in approximately 58% of patients [4]. Continued follow‐up of CoC‐HRA cases will help determine whether comparable long‐term outcomes can be achieved.

## 5 Conclusion

In conclusion, both resurfacing procedures restored gait function and activity to higher levels than THA. The findings demonstrated that both CoC‐HRA and MoM‐HRA patients have similar postoperative functional outcomes, comparable to healthy controls that THA did not. CoC‐HRA may be a suitable alternative to MoM‐HRA for patients with hip OA seeking an active lifestyle, addressing metal‐on‐metal complications while maintaining the functional benefits observed in MoM‐HRA. For hip OA patients where MoM‐HRA has been contraindicated, such as woman and men with smaller femoral head sizes, CoC‐HRA may provide a suitable option to these currently excluded patients.

## Conflicts of Interest

Justin Cobb is a paid employee of Embody Orthopeadics Limited. The remaining authors declare no conflicts of interest.
